# Cell Proliferation Pattern and *Twist* Expression in an Aplacophoran Mollusk Argue Against Segmented Ancestry of Mollusca

**DOI:** 10.1002/jez.b.22714

**Published:** 2016-12-14

**Authors:** Emanuel Redl, Maik Scherholz, Tim Wollesen, Christiane Todt, Andreas Wanninger

**Affiliations:** ^1^ Faculty of Life SciencesDepartment of Integrative ZoologyUniversity of ViennaViennaAustria; ^2^ University Museum, The Natural History CollectionsUniversity of BergenBergenNorway

## Abstract

The study of aplacophoran mollusks (i.e., Solenogastres or Neomeniomorpha and Caudofoveata or Chaetodermomorpha) has traditionally been regarded as crucial for reconstructing the morphology of the last common ancestor of the Mollusca. Since their proposed close relatives, the Polyplacophora, show a distinct seriality in certain organ systems, the aplacophorans are also in the focus of attention with regard to the question of a potential segmented ancestry of mollusks. To contribute to this question, we investigated cell proliferation patterns and the expression of the *twist* ortholog during larval development in solenogasters. In advanced to late larvae, during the outgrowth of the trunk, a pair of longitudinal bands of proliferating cells is found subepithelially in a lateral to ventrolateral position. These bands elongate during subsequent development as the trunk grows longer. Likewise, expression of *twist* occurs in two laterally positioned, subepithelial longitudinal stripes in advanced larvae. Both, the pattern of proliferating cells and the expression domain of *twist* demonstrate the existence of extensive and long‐lived mesodermal bands in a worm‐shaped aculiferan, a situation which is similar to annelids but in stark contrast to conchiferans, where the mesodermal bands are usually rudimentary and ephemeral. Yet, in contrast to annelids, neither the bands of proliferating cells nor the *twist* expression domain show a separation into distinct serial subunits, which clearly argues against a segmented ancestry of mollusks. Furthermore, the lack of *twist* expression during the development of the ventromedian muscle argues against homology of a ventromedian longitudinal muscle in protostomes with the notochord of chordates.

## INTRODUCTION

Seriality, that is, a repetitive arrangement of morphological structures along the anterior–posterior axis, is a common feature across the Metazoa. However, only few taxa include representatives that possess a concerted seriality of several organ systems with involvement of the mesoderm, a situation, which is often called segmentation *sensu stricto*, or metamerism. These taxa, which include the Annelida, Kinorhyncha, Panarthropoda, and Chordata, occupy very distant positions on the tree of life, with the Annelida belonging to the Lophotrochozoa, the Kinorhyncha and the Panarthropoda to the Ecdysozoa, and the Chordata to the Deuterostomia, respectively. Despite their phylogenetic distance, similarities do exist in the way as to how some representatives of these groups form their “segments”, including the expression domains of several so‐called “segmentation genes”, that is, genes that are involved in the process of building a segmented body (Balavoine and Adoutte, 2003; Seaver, 2003; Tautz, 2004; Blair, 2008; Couso, 2009; Chipman, 2010). This has led to the notion that segmentation or metamerism was already present in the last common ancestor (LCA) of the Bilateria and that it was reduced or lost in all nonsegmented lineages (e.g., Balavoine and Adoutte, 2003; Couso, 2009). The competing hypothesis assumes an independent evolution of segmentation in the above‐mentioned taxa (e.g., Erwin and Davidson, 2002; Chipman, 2010). These two hypotheses differ fundamentally in their approach to the concept of homology, especially its interconnection between the different hierarchical levels of biological organization. The first scenario assumes that homology of gene expression patterns and eventually the resulting morphological structures can be inferred solely from the homology of the genes involved, that is, that a similar expression pattern or structure is always homologous if the genes that are involved in its formation are. However, examples are known where homologous morphological structures are built by nonhomologous genes (a phenomenon commonly known as developmental systems drift) and, on the contrary, homologous genes may be involved in the formation of nonhomologous structures. Consequently, homology actually has to be assessed separately on different levels of biological organization, such as genes, gene expression patterns, developmental origin, and morphological features, thus calling for multilevel assessments of potential homology of segments across the bilateria (for reviews see, e.g., Abouheif, 1997; Abouheif et al., 1997; Wray and Abouheif, 1998; Minelli and Fusco, 2013; Wagner, 2014, 2015; Minelli, 2015).

The only unambiguously segmented lophotrochozoans are the Annelida and, despite some variation, they usually build the majority of their segments in a unique way from a posterior growth zone in an anterior to posterior progression (Anderson, 1966, 1973; Shimizu and Nakamoto, 2001; De Rosa et al., 2005; Seaver et al., 2005; Fischer et al., 2010; Balavoine, 2014). Although some molluscan subtaxa, such as the Polyplacophora and Monoplacophora, exhibit a pronounced seriality in certain organ systems (e.g., neuromuscular subsets, shell plates, ctenidia), developmental studies failed to recover any rudiment of an annelid‐like formation pattern (Friedrich et al., 2002; Voronezhskaya et al., 2002; Wanninger and Haszprunar, 2002). This argues against a segmented ancestry of mollusks, thus also rendering a segmented LCA of the entire Lophotrochozoa more unlikely. On the other hand, rudiments of ancestral segmentation have been found in lophotrochozoans that are not segmented as adults, such as echiurans and sipunculans. In these two annelid taxa typical traits of annelid‐like segmentation, such as segmentally arranged perikarya or a posterior proliferation zone, occur during ontogeny (Hessling, 2002, 2003; Hessling and Westheide, 2002; Kristof et al., 2008, 2011; Wanninger et al., 2009).

A promising approach to test for cryptic segmentation is to assess cell proliferation and gene expression patterns during development. To this end, it has been shown that in certain stages of annelid development the presence of segments and/or a posterior growth zone is mirrored by the pattern of proliferating cells, even in those representatives that have lost morphological segmentation such as the above‐mentioned echiurans and sipunculans (Hessling, 2003; de Rosa et al., 2005; Seaver et al., 2005; Brinkmann and Wanninger, 2010; Kristof et al., 2011). The transcription factor *twist*, which is generally expressed in mesoderm progenitor or mesodermal cells during specification and/or differentiation of the third germ layer in bilaterians (Sommer and Tautz, 1994; Castanon and Baylies, 2002; Technau and Scholz, 2003), is a promising marker for a potential segmental mode of development. This is because in all segmented animals investigated so far, *twist* is expressed during certain stages of development in a series of stripes or patches along the anterior–posterior axis (e.g., Tavares et al., 2001; Handel et al., 2005; Yamazaki et al., 2005; Dill et al., 2007; Price and Patel, 2008; Pfeifer et al., 2013; Kozin et al., 2016).

With regard to mollusks, the study of the vermiform aplacophorans (i.e., Solenogastres or Neomeniomorpha and Caudofoveata or Chaetodermomorpha) has traditionally been regarded as crucial for reconstructing the morphology of the LCA of the phylum (see, e.g., Salvini‐Plawen, 1972, 2003). Together with the fact that recent studies suggest a sister‐group relationship of aplacophorans and polyplacophorans (Kocot et al., 2011; Smith et al., 2011, 2013; Vinther et al., 2012), this places them in the focus of attention when it comes to the question of a potentially segmented ancestry of mollusks. Thus, we investigated cell proliferation patterns and the expression of the mesodermal marker gene *twist* during larval development in two species of solenogasters, *Wirenia argentea* Odhner, 1921 and *Gymnomenia pellucida* Odhner, 1921.

## MATERIALS AND METHODS

### Animal Cultures

Specimens of *W. argentea* and *G. pellucida* were collected, maintained, and reared from January to May 2012, November 2012 to February 2013, and November to December 2013, respectively, as described in Redl et al. (2014) with the following modifications during the last season. The sieved fraction of each sediment sample was kept in 20 μm filtered and UV‐sterilized sea water with a salinity of 35‰ (FSSW) precooled to 4°C rather than in deep water from the sampling location and every 4 days, rather than changing a part of the water, the adult specimens were transferred to new plastic jars with fresh FSSW, which increased egg laying productivity. Furthermore, in *W. argentea*, the newly laid eggs, rather than the newly hatched larvae, were isolated from the cultures, put into a separate jar with FSSW, and kept under the same conditions as the adults. Freshly hatched larvae were then isolated from this jar and kept under the same conditions. The age of the larvae is given in days posthatching (dph), whereby 0–1 dph is used for larvae ranging from newly hatched to an age of 24 hr.

Voucher specimens of adult animals of both species from an earlier collection at the locality in Hauglandsosen (see Redl et al., 2014) are deposited in the Natural History Collections of the University Museum of Bergen, Norway (Collection numbers: ZMBN 94730 for *W. argentea*, ZMBN 94742–94744 for *G. pellucida*). Barcoding data for these specimens are available in the Barcode of Life Data System (BOLD) (Ratnasingham and Hebert, 2007) under the following BOLD IDs: UM_NB_aplac76 for *W. argentea* and UM_NB_aplac88‐90 for *G. pellucida*.

### 5‐Ethynyl‐2′‐deoxyuridine Labeling and Analysis

5‐Ethynyl‐2′‐deoxyuridine (EdU) is a thymidine analogue, which is incorporated into DNA during DNA synthesis and can thus be used to label proliferating cells (Cavanagh et al., 2011). EdU labeling was done using a Click‐iT EdU Alexa Fluor 488 Flow Cytometry Assay Kit (Molecular Probes, Life Technologies, Carlsbad, CA). Larvae of *W*. *argentea* with an age of 7–17 dph were incubated alive for 6 hr at 7°C in an 8 μM solution of EdU (Component A) in FSSW. Larvae of *G*. *pellucida* with an age of 8–19 dph were incubated alive for 24 hr at 7°C in a 5 μM solution of EdU in FSSW. After incubation, all larvae were relaxed for 20–25 min at 4°C by adding a 3.2% magnesium chloride solution and subsequently fixed for 1.5 hr at room temperature (RT) with 4% paraformaldehyde (PFA) in 0.1 M phosphate buffer (PB, pH 7.3). The samples were then rinsed four times in 0.1 M PB (pH 7.3) with 0.1% sodium azide at RT for a total period of 45 min. They were stored in PB with 0.1% sodium azide at 4°C. For negative controls, additional larvae of both species were fixed identically but without prior EdU incubation and consequently rendered no signal.

All larvae were decalcified for 1 hr at RT in 0.05 M EGTA (pH 7.2) and rinsed three times at 4°C in PB for a total period of 3.5 hr (*W. argentea*) or 19 hr (*G. pellucida*), respectively. They were then incubated in 1× Click‐iT saponin‐based permeabilization and wash reagent (Component E) in a solution of 1% bovine serum albumin (Albumin Fraction V, US origin; Carl Roth, Karlsruhe, Germany) in PB (permeabilization and wash reagent, PWR) for 19 hr (*W. argentea*) or for 24 hr (*G. pellucida*) at 4°C. Subsequently, each sample (encompassing between 5 and 17 larvae) was incubated for 24–25 hr at 4°C in 2 mL of reaction cocktail consisting of 10 μL working solution of Alexa Fluor 488 azide (component B) in dimethylsulfoxide (component C), 40 μL of 0.1 M aqueous solution of copper (II) sulfate (component F), 200 μL of 1× Click‐iT EdU buffer additive (component G) in deionized water, and 1.75 mL of PB (the treatment with the reaction cocktail and all subsequent steps were done in the dark). Hereupon, larvae of *G. pellucida* were rinsed three times for 15 min each at 4°C in PWR (whereby in the first washing step, the solution contained DAPI (Molecular Probes, Life Technologies) at a concentration of 2.5 μg/mL), mounted in Fluoromount G (SouthernBiotech, Birmingham, AL) on microscope slides, and stored at 4°C in the dark until examination.

Larvae of *W. argentea* were rinsed three times for 15 min each at 4°C in PWR. Then, a solution of rabbit anti‐serotonin (5‐HT; polyclonal; Sigma‐Aldrich, St. Louis, MO, or ImmunoStar, Hudson, WI) and mouse antiacetylated α‐tubulin (monoclonal; Sigma‐Aldrich) primary antibodies in PWR was applied for 24 hr at 4°C, whereby each antibody had a dilution of 1:600 (larvae for negative controls were treated with PWR without primary antibodies and rendered no signal). The larvae were then rinsed three times for 15 min each at 4°C in PWR and subsequently incubated for 25 hr at 4°C in a solution of Alexa Fluor 568 goat anti‐mouse and Alexa Fluor 633 goat anti‐rabbit secondary antibodies (both from Molecular Probes, Life Technologies) in PWR with each antibody in a dilution of 1:300. Finally, the larvae were rinsed three times at 4°C in PWR for a total period ranging between 45 min and 1.5 hr, mounted in Fluoromount‐G (Southern Biotech) on microscope slides, and stored at 4°C in the dark until examination.

The analysis was done using a Leica TCS SP5 II confocal laser scanning microscope equipped with the software Leica Application Suite Advanced Fluorescence (LAS AF), Version 2.6.0–2.6.3 (Leica Microsystems, Wetzlar, Germany). Approximately 80 specimens were investigated in total. The obtained image data were further analyzed and processed with the LAS AF Lite software, Version 3.3.0, as well as with Imaris x64, Version 7.3.1 (Bitplane, Zurich, Switzerland), Adobe Photoshop CS5 Extended, Version 12.0 to 12.0.4 × 64, and Adobe Photoshop CS6 Extended, Version 13.0.1 × 64 (Adobe Systems, San José, CA). The schematic drawings were generated with Adobe Illustrator CS5, Version 15.0.0 and Adobe Illustrator CC 2015, Version 1.0 (Adobe Systems).

### Gene Expression Data and Analysis

From RNA extraction until the end of the *in situ* hybridization protocol, all steps were conducted using nuclease‐free (or diethylpyrocarbonate‐treated) water.

#### Transcriptome Data, Cloning, and Probe Synthesis

For sequencing of the transcriptome, total RNA was extracted from larvae of *W. argentea* using a Qiagen RNeasy Mini Kit (Qiagen, Venlo, Netherlands) with the QIAshredder homogenizer (Qiagen). The larval material used was either shock frozen on dry ice immediately before RNA extraction or conserved in RNAlater. Additionally, RNA was extracted from adult specimens using TRI reagent (Sigma‐Aldrich) according to the manufacturer's instructions with the optional centrifugation step after homogenization and the following modifications. The animals were shock frozen with dry ice immediately before homogenization and RNA precipitation was performed with a 1:1 mixture of isopropanol and a high salt precipitation solution containing 0.8 mol/L trisodium citrate dihydrate and 1.2 mol/L sodium chloride. All extracted RNA samples were redissolved in water and stored at –80°C. Larval and adult RNA samples were pooled, used for the preparation of an amplified short insert cDNA library (150–250 bp insert size), and sequenced by Illumina technology by the company Eurofins (Ebersberg, Germany). The library (Kit version TruSeq SBS Kit v3) was sequenced together with another two bar‐coded libraries in one channel of HiSeq 2000 with Illumina chemistry v3.0. Sequences were demultiplexed according to the 6 bp index code with 0 mismatch allowed. In both cases, a PhiX library was added before sequencing to estimate the error rate of the sequences. Preprocessing of the resulting paired‐end libraries was carried out using the multithreaded command line tool Trimmomatic, Version 0.3.2 (Bolger et al., 2014). The known specific Illumina adapters were removed with the following parameter: “ILLUMINACLIP:adapters/TruSeq3‐PE‐2.fa:2:30” and the filtering by quality and length was executed with the following command line: “SLIDINGWINDOW:4:20 MINLEN:40”. The quality of the filtered libraries was assessed with the software fastx_toolkit (http://hannonlab.cshl.edu/fastx_toolkit) with respect to the quality score of the bases, the GC‐content, and the read length. The filtered transcriptome datasets were reconstructed into contiguous cDNA sequences with IDBA‐tran, Version 1.1.1 (Peng et al., 2013) with the parameters “–mink 2 –maxk 60 –step 5 –max_count 3”. The quantitative quality assessment of the reconstructed datasets regarding the number of transcripts, number of total bases reconstructed, N50 value, and GC content was carried out using the software QUAST, Version 2.3 (Gurevich et al., 2013).

The assembly was analyzed and mined for the *twist* ortholog using published amino acid and nucleotide sequence data from the databases of the National Center for Biotechnology Information (NCBI, Bethesda, MD; www.ncbi.nlm.nhi.gov) together with the BLAST algorithm and the software Geneious, Version 6.1.6 (Biomatters, Auckland, New Zealand). The longest and best‐fitting contig from the assembly was chosen and primers with the following sequences were designed for amplifying a part of 705 nucleotides length spanning the conserved region of the gene: forward: 5′‐CATTCTGGCACCAATCCTACCAAATAC‐3′ (predicted annealing temperature: 62.3°C), reverse: 5′‐CGCATGTCTATTTGTCGTTCATGATTG‐3′ (predicted annealing temperature: 61.7°C). Primers were synthesized by Invitrogen, Life Technologies. The nucleotide sequence of the whole contig as well as the translated amino acid sequence of the coding region are available from the NCBI databases (Accession number: KY034417).

For the first‐strand cDNA synthesis, total RNA was extracted from live larvae and adults using a Qiagen RNeasy Mini Kit with the QIAshredder homogenizer. Larval and adult RNA samples were pooled before cDNA synthesis, which was done with a 1st Strand cDNA Synthesis Kit for RT‐PCR (AMV) (Roche, Basel, Switzerland) using the Oligo‐p(dT)_15_ Primers (the RNA sample was denatured for 15 min at 65°C and placed on ice for 5 min before being added to the reaction; no gelatin and a‐^32^P dCTP were used). The resulting cDNA sample was diluted 1:50 with water. From this cDNA as a template, the above‐mentioned 705 nucleotide part of the *W*. *argentea twist* ortholog was amplified via touchdown PCR (temperature range: 63–59°C) using the GoTaq Flexi DNA Polymerase reagents (Promega, Madison, WI) with the corresponding PCR nucleotide mix (Promega). The PCR product was subjected to a gel electrophoresis with a 2% agarose gel in 1× TAE buffer (Carl Roth), the band with the expected size was excised, and DNA was extracted using a QIAquick Gel Extraction Kit (Qiagen). Ligation of the insert into the plasmid was carried out overnight at 4°C using the pGEM‐T Easy Vector System I (Promega). The plasmids were then used to transform *E. coli* JM109 Competent Cells (Promega), which were subsequently grown on LB‐Agar plates. Transformed bacteria were selected, grown in Minipreps, and plasmid DNA was extracted using the QIAprep Spin Miniprep Kit (Qiagen) (the DNA was eluted using the provided Buffer EB). A DNA sample was then sequenced by the company Microsynth (Vienna, Austria) and the obtained sequence data were compared to published *twist* sequence data from the NCBI databases and to the assembled transcriptome using the BLAST algorithm and Geneious, Version 6.1.6. The insert of the plasmids was amplified via a standard PCR using the GoTaq Flexi DNA Polymerase reagents with the corresponding PCR nucleotide mix and M13 forward (5′‐GTTTTCCCAGTCACGACGTT‐3′; annealing temperature: 60°C) and reverse (5′‐GACCATGATTACGCCAAGCTA‐3′; annealing temperature: 60°C) primers. The amplified inserts were purified using a GeneJET PCR Purification Kit (Thermo Fisher Scientific, Waltham, MA) and subsequently used as a template for antisense RNA probe synthesis, which was done with a DIG RNA Labeling Mix, 10× concentration, and SP6 RNA polymerase with the corresponding transcription buffer (all from Roche). Two microliters of 100 mM dithiothreitol were added to the transcription reaction and, after the reaction, template DNA was removed by incubation with DNase I, RNase free (Roche). Precipitation of RNA was done at –80°C. One microliter of Protector RNase Inhibitor (Roche) was added after dissolving the RNA pellet in water. The RNA probes were stored at –80°C until usage.

#### In Situ Hybridization

Developmental stages of *W. argentea* were fixed with 4% PFA in 0.1 M 3‐(*N*‐morpholino)propanesulfonic acid buffer (with 0.5 M/L sodium chloride, 2 mM/L magnesium sulfate, and 1 mM/L EGTA added) for 45 min at RT. Specimens from 8 dph onward were relaxed prior to fixation for 20 to 30 min at 4°C by adding a 3.2% magnesium chloride solution. After fixation, the animals were stepped into ethanol by dropwise addition of precooled (+4°C) or prechilled (–20°C) 75% ethanol, washed three times for a total period of 15–30 min in precooled (+4°C) or for 45 min to 1.5 hr in prechilled (–20°C) 75% ethanol, and stored in fresh 75% ethanol at –20°C.

For *in situ* hybridizations, the samples were first stepped into 4% PFA in 1× Roti‐Stock phosphate buffered saline (PBS; pH 7.4; Carl Roth) with 0.05 M/L EGTA (PPE) and decalcified in PPE for 1 hr at RT. They were then washed six times for 5 min each at RT in PBS with 0.1% Tween 20 (phosphate buffered saline with Tween 20, PBT; Carl Roth) and warmed up to 37°C in a water bath during the last washing step. Enzyme treatment was done with a solution of 10 μg/mL Proteinase K (Roche) in PBT for 10 min at 37°C without agitation. After that, the specimens were washed twice for 5 min each at RT in PBT, twice for 5 min each in 1% triethanolamine (TEA) in PBT, two to four times for 5 min each in instantly made 0.3% acetic anhydride and 1% TEA in PBT, and again twice for 5 min each in PBT. Then, the samples were postfixed in 4% PFA in PBS for 45 min at RT and rinsed again five times for 5 min each at RT in PBT. They were subsequently stepped into the hybridization buffer (HB) consisting of 50% formamide with 0.075 M/L trisodium citrate, 0.75 M/L sodium chloride, 5 mM/L EDTA, 50 μg/mL heparin sodium salt (Sigma‐Aldrich), 1× Denhardt's Solution (Carl Roth), 100 μg/mL RNA from torula yeast, Type VI (Sigma‐Aldrich), and 5% dextran sulfate sodium salt from *Leuconostoc* spp. (Sigma‐Aldrich). The samples were then put into new HB, warmed up to 56–59°C in a water bath, and prehybridized at that temperature for 15–20 hr. RNA probes were diluted in HB to a final concentration of 1–2 μg/mL, denatured for 10 min at 85°C, and applied to the samples. Hybridization was performed for 24–26 hr at 56–59°C. Afterwards, the specimens were kept at the hybridization temperature and rinsed three times for 20 min each in 50% formamide with 0.06 M/L trisodium citrate, 0.6 M/L sodium chloride, and 0.1% Tween 20, twice for 20 min each in 50% formamide with 0.03 M/L trisodium citrate, 0.3 M/L sodium chloride, and 0.1% Tween 20, and three times for 15 min each in 50% formamide with 0.015 M/L trisodium citrate, 0.15 M/L sodium chloride, and 0.1% Tween 20. They were then put at RT to cool down before being stepped into and washed three times for 20 min each at RT in 0.015 M trisodium citrate solution with 0.15 M/L sodium chloride (pH 7) and 0.1% Tween 20. Subsequently, the animals were stepped into and washed three times for 5 min each at RT in 0.1 M maleic acid buffer (MAB) with 0.15 M/L sodium chloride and 0.1% Tween 20 (pH 7.5; MAB). Blocking of unspecific binding sites was done for 3 hr at RT with a 2% solution of Blocking Reagent (Roche; Product No. 11096176001) in MAB (2% block). Anti‐digoxigenin (DIG)‐AP, Fab fragments (Roche) were applied in a 1:2,500 to 1:5,000 dilution in 2% block for 13–16 hr at 4°C. The samples were then washed eight times for 20 min each at RT in PBT, twice for 5 min each without agitation in 0.1 M Tris buffer with 0.1 M/L sodium chloride (pH 9.5; AP buffer) and 0.1% Tween 20, and twice for 10 min each without agitation in AP buffer with 50 mM/L magnesium chloride and 0.1% Tween 20. Finally, the specimens were transferred into the staining buffer consisting of AP buffer with 50 mM/L magnesium chloride, 7.5% polyvinyl alcohol, and 20 μL/mL NBT/BCIP Stock Solution (Roche) and color was developed at 4°C for 3.5–30 hr.

The samples were then rinsed at RT either four times for at least 5 min each in PBT or twice for 5 min each in 0.1 M glycine in PBT (pH 2.2) and twice for at least 5 min each in PBT. They were then fixed for 12–26 hr at 4°C in 4% PFA in PBS, subsequently rinsed at RT four times for at least 5 min each in PBT, and finally stored in PBT at 4°C until examination.

#### Mounting and Clearing

Uncleared specimens were mounted in glycerol, Fluoromount‐G (SouthernBiotech), or PBS on microscope slides. For clearing, which was done at RT, specimens were first stepped into deionized water, washed four times for 5 min each in deionized water, stepped into 100% ethanol, washed three times for 5 min each in 100% ethanol, transferred into a mixture of benzyl benzoate and benzyl alcohol in a ratio of 1:1 or 2:1, and mounted in the same mixture.

#### Microscopy, Three‐Dimensional Rendering, and Image Processing

The slides were analyzed and light micrographs were taken on a Nikon SMZ25 stereo microscope equipped with a Nikon Digital Sight DS‐Ri1 camera and the software NIS‐Elements BR, Version 4.30.02 64bit (Nikon Corporation, Shinagawa, Tokyo, Japan), and on an Olympus BX53 microscope equipped with an Olympus DP73 camera and the software cellSens Standard, Version 1.11 (Olympus Corporation, Shinjuku, Tokyo, Japan). Confocal scans were conducted using a Leica TCS SP5 II confocal laser scanning microscope equipped with LAS AF, Version 2.6.0–2.6.3. A 405 nm laser was used for autofluorescence signal of the larvae in fluorescence mode and a 633 nm laser was used to scan the gene expression signal using the reflection mode (compare Jékely and Arendt, 2007; Fritsch et al., 2015). The obtained image data were further analyzed and processed with LAS AF Lite, Version 3.3.0. From the confocal image stacks, three‐dimensional (3D) renderings were produced and processed with Imaris x64, Version 7.3.1. All images were finally processed with Adobe Photoshop CS5 Extended, Version 12.0 to 12.0.4 × 64, Adobe Photoshop CS6 Extended, Version 13.0.1 × 64, and Adobe Photoshop CC 2015 (Adobe Systems). The schematic drawings were generated with Adobe Illustrator CS5, Version 15.0.0 and Adobe Illustrator CC 2015, Version 1.0.

Approximately 130 specimens were processed and investigated in total and approximately 25 of them were scanned.

### Bioinformatics Analysis of the *Twist* Ortholog of *W. argentea*

Several twist, paraxis, and dHAND amino acid sequences were downloaded from the NCBI databases (see Supplementary Fig. S1). All sequences were aligned with the amino acid sequence of the contig from the assembled transcriptome, which was used to design the primers for the *twist* probe, using the ClustalW algorithm (cost matrix: BLOSUM; gap open cost: 10; gap extend cost: 0.1). The alignment was manually trimmed to a region that encompasses all the conserved regions and lies within the boundaries of the cloned fragment. A consensus tree was then calculated from this alignment using the neighbor‐joining (genetic distance model: Jukes–Cantor) and the bootstrap algorithm (number of replicates: 10,000; support threshold: 50%). The tree was rooted using the paraxis + dHAND cluster as outgroup. The whole bioinformatics analysis was performed with Geneious, Version 6.1.6, and the graphics were enhanced with Adobe Photoshop CC 2015.

## RESULTS

### Cell Proliferation Patterns in *W. argentea* and *G. pellucida*

**Figure 1:**
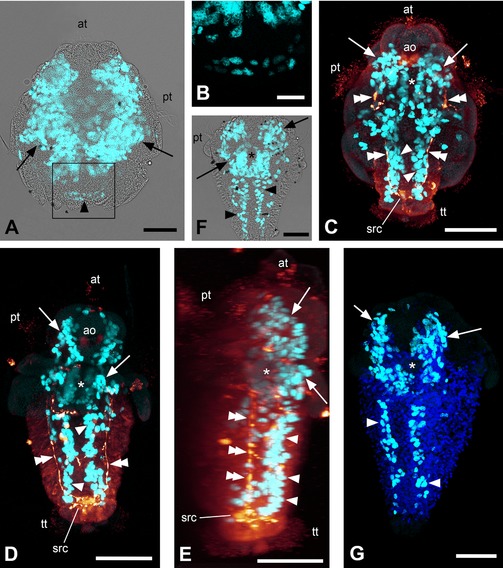
EdU and immunocytochemical labeling of solenogaster developmental stages. Maximum intensity projections of confocal image stacks; apical/anterior is up in all panels; scale bar equals 50 μm in (**A**) and (**C**–**G**) and 20 μm in (**B**). (**A**) Labeling of nuclei of proliferating cells (light blue) with overlay of transmitted light image; early larva of *Gymnomenia pellucida* showing kidney‐shaped apical proliferation zones (arrows) and anlage of longitudinal bands of proliferating cells (arrowhead). (**B**) Detail of boxed area in (**A**) without overlay of transmitted light image. (**C**) Labeling of nuclei of proliferating cells (light blue) and serotonin‐like immunoreactive (LIR) components of the nervous system (orange); 12–13 days posthatching (dph) larva of *Wirenia argentea* scanned in ventral aspect showing anterior proliferation zones (arrows) with gap in the region of the foregut (asterisk), longitudinal bands of proliferating cells (arrowheads), and longitudinal (lateral) neurite bundles (double arrowheads). (**D**) Labeling of nuclei of proliferating cells (light blue) and serotonin‐LIR components of the nervous system (orange); 16–17 dph larva of *W*. *argentea* scanned in ventral aspect showing anterior proliferation zones (arrows) with gap in the region of the foregut (asterisk), longitudinal bands of proliferating cells (arrowheads), and longitudinal (lateral) neurite bundles (double arrowheads). (**E**) Labeling of nuclei of proliferating cells (light blue) and serotonin‐LIR components of the nervous system (orange); 3D reconstruction (volume rendering, MIP (max) mode) of the same confocal image stack as in (**D**) in right lateral view. Note that the lateral neurite bundles (double arrowheads) are located dorsally to the longitudinal bands of proliferating cells (arrowheads). Serotonin‐LIR elements at the same level as the longitudinal bands of proliferating cells belong to the developing ventral nervous system. The asterisk marks the region of the foregut and arrows point to the anterior proliferation zones. (**F)** Labeling of nuclei of proliferating cells (light blue) with overlay of transmitted light image; late larva of *G. pellucida* scanned in ventral aspect showing anterior proliferation zones (arrows) with gap in the region of the foregut (asterisk) and longitudinal bands of proliferating cells (arrowheads). (**G**) Labeling of nuclei of proliferating cells (light blue) and cell nuclei with DAPI (dark blue); late larva of *G. pellucida* scanned in left ventrolateral aspect showing anterior proliferation zones (arrows) with gap in the region of the foregut (asterisk) and longitudinal bands of proliferating cells (arrowheads). ao, apical organ; at, apical tuft; pt, prototroch; src, suprarectal commissure; tt, telotroch.

**Figure 2:**
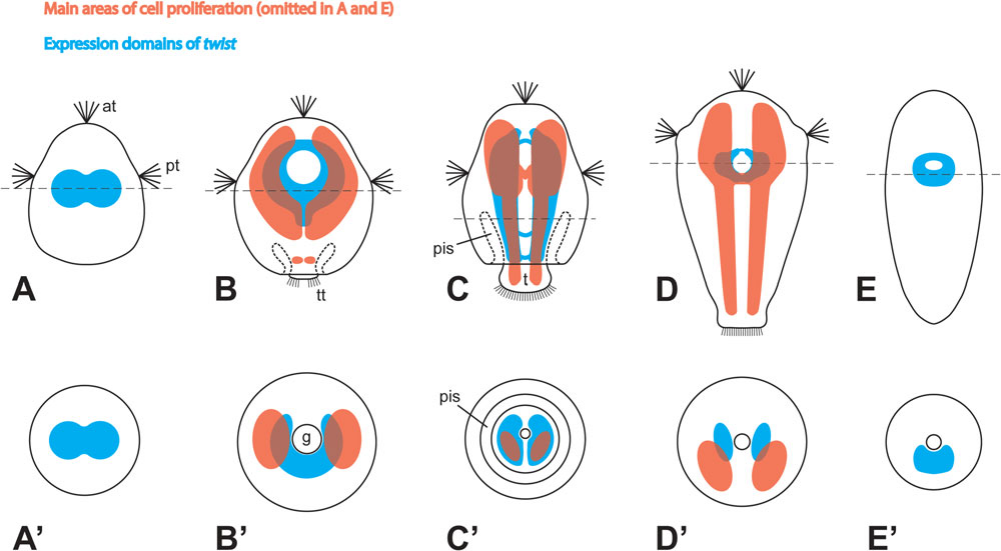
Schematic summary of cell proliferation pattern and *twist* expression in Solenogastres. Apical/anterior is up in (**A**–**E**); dorsal is up in (**A**′–**E**′). Note that cell proliferation areas are not shown in (**A**) and (**E**) because no EdU labeling data are available for these stages. In stages where *twist* expression was not fully consistent, the maximum expression domain is depicted. (**A–D**) Ventral views of larvae. (E) Ventral view of juvenile. (**A**′–**E**′) Cross‐sections of corresponding stages at the position of the dashed lines in (**A**–**E**). at, apical tuft; pis, periimaginal space (*sensu* Salvini‐Plawen, 1980), that is, the cavity between the calymma and the outgrowing trunk of the animal; g, gut; pt, prototroch; t, trunk; tt, telotroch.

In early larvae, cells proliferate at the highest rate in a pair of often kidney‐shaped areas, which run along most of the longitudinal axis and encompass the area where the foregut is about to be formed (Figs. 1A and 2B and B′). Additionally, cell divisions can be observed at the posterior end of the larvae, which mark the beginning of the outgrowth of the trunk (Figs. 1A and B and 2B). This area subsequently elongates together with the trunk to form two continuous lateral to ventrolateral rows of proliferating cells. These longitudinal bands never show any sign of separation into distinct serial subunits (Figs. 1C–G and 2C, C′, D, and D′). The above‐mentioned kidney‐shaped areas persist in what is now the anteriormost part of the larva. Between their posterior parts, an additional area of high cell proliferation can be observed around the foregut (Figs. 1C–G and 2C and D). The areas of highest cell proliferation are situated mostly subepithelially and are positioned predominantly in the ventral half of the larva (Figs. 1E and 2C′ and D′).

In *W. argentea*, the longitudinal bands of proliferating cells are first situated in a lateral to ventrolateral position encompassing the region of the developing lateral nerve cords. With the development of the ventral nervous system, the bands shift to the region of the developing ventral nerve cords, that is, to a purely ventrolateral position, and are thus situated ventromedially to the developing lateral nerve cords (Fig. 1E).

### Gene Orthology Assessment

The alignment demonstrates the presence of the bHLH domain and the WR motif characteristic for twist in the partial amino acid sequence of the contig from the assembled transcriptome, which was used to design the primers for the *twist* probe, demonstrating that the contig sequence is actually a part of the *twist* ortholog of *W*. *argentea* (Supplementary Fig. S1A; Spring et al., 2000). Furthermore, the tree shows three distinctive clusters with high support values, one for twist and one for each of the other two bHLH transcription factors, paraxis and dHAND, substantiating this result (Supplementary Fig. S1B).

### Expression of the *Twist* Ortholog in *W. argentea*

**Figure 3:**
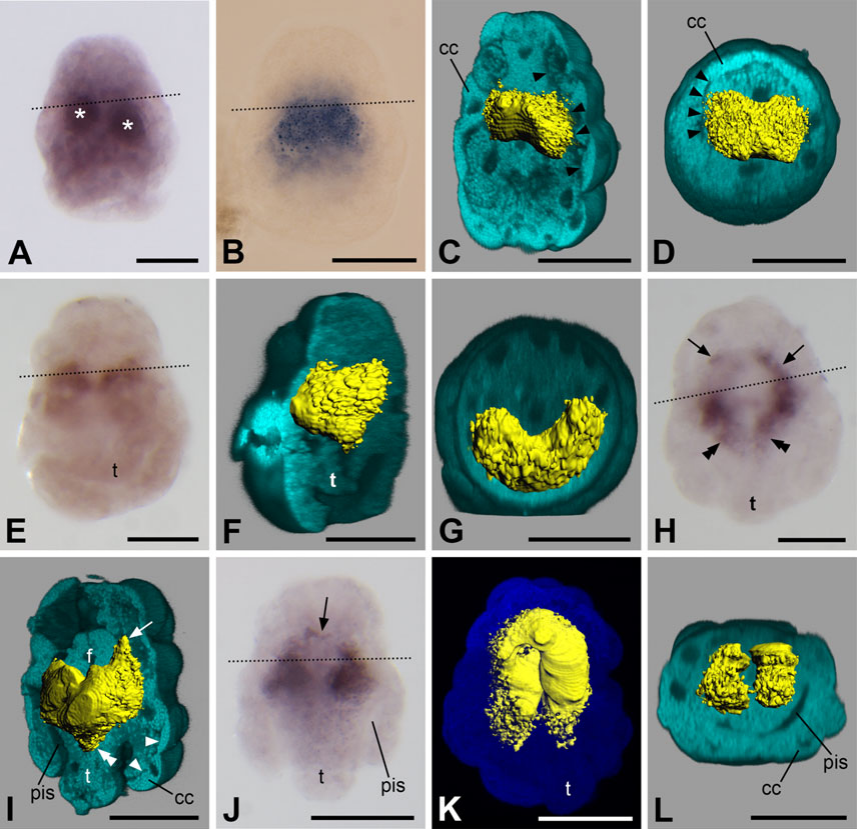
Expression of *twist* in larvae of *Wirenia argentea*. Apical/anterior is up in (**A**–**C**), (**E**), (**F)**, and (**H**–**K**); dorsal is up in (**D**) and (**G**); ventral is up in (**L**); scale bar equals 50 μm in all panels; dotted lines indicate the region of the prototroch. (**A**) 0–1 days posthatching (dph) larva showing a pair of spherical regions with *twist* expression (asterisks). (**B**) 0–1 dph larva with peanut‐shaped *twist* expression domain. (**C**) 3D reconstruction of a confocal scan with volume rendering (blend mode) of autofluorescence of larva (light blue) and surface rendering of reflection signal of expression domain (yellow); 0–1 dph larva in left ventrolateral view with ventral part of autofluorescence signal omitted. Note that the gene expression signal does not extend to the outermost cell layer of the larva, the borders of which are well visible (arrowheads). (**D**) Same specimen and reconstruction as in (**C**) in apical view with apical part of autofluorescence signal omitted. Note that the gene expression signal does not extend to the outermost cell layer of the larva, the borders of which are well visible (arrowheads). (**E**) 2–3 dph larva. (**F**) 3D reconstruction of a confocal scan with volume rendering (blend mode) of autofluorescence of larva (light blue) and surface rendering of reflection signal of expression domain (yellow); same specimen as in (**E**) in left ventrolateral view with left part of autofluorescence signal omitted. Note that the gene expression signal does not extend to the outermost cell layer of the larva. (**G**) Same specimen and reconstruction as in (**F**) in apical view with apical part of autofluorescence signal omitted. Note that the gene expression signal does not extend to the outermost cell layer of the larva. (**H**) 6–7 dph larva showing apical/anterior (arrows) and abapical/posterior (double arrowheads) elongations of the gene expression domain when compared to previous stages. (**I**) 3D reconstruction of a confocal scan with volume rendering (blend mode) of autofluorescence of larva (light blue) and surface rendering of reflection signal of expression domain (yellow); same specimen as in (**H**) in left ventrolateral view with ventral part of autofluorescence signal omitted. Note the apical/anterior (arrows) and abapical/posterior (double arrowheads) extension of the gene expression domain when compared to previous stages. The gene expression signal does not extend to the outermost cell layer of the larva, the borders of which are well visible (arrowheads). (**J**) 6–7 dph larva showing anterior extension of gene expression domain surrounding the developing foregut (arrow). (**K**) 3D reconstruction of a confocal scan with volume rendering (MIP (max) mode) of autofluorescence of larva (dark blue) and surface rendering of reflection signal of expression domain (yellow); 6–7 dph larva in ventral view. (**L**) 3D reconstruction of a confocal scan with volume rendering (blend mode) of autofluorescence of larva (light blue) and surface rendering of reflection signal of expression domain (yellow); same specimen as in (**K**) in posterior view with posterior part of autofluorescence signal omitted. Note that the gene expression signal is only present in the trunk and not in the calymma. pis, peri‐imaginal space; cc, calymma cell; f, developing foregut; t, outgrowing trunk.

**Figure 4:**
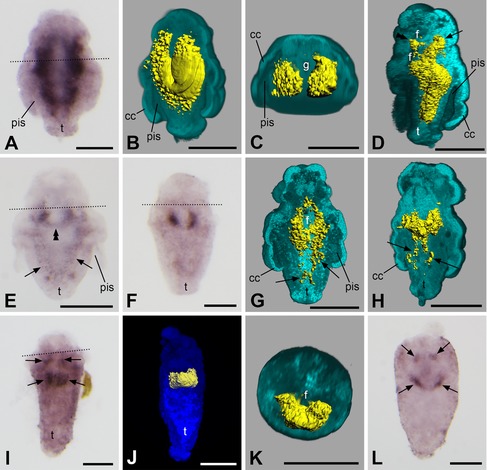
Expression of *twist* in larvae and juveniles of *Wirenia argentea*. Apical/anterior is up in (**A**), (**B**), (**D**–**J**), and (**L**); dorsal is up in (**C**) and (**K**); scale bar equals 50 μm in all panels; dotted lines indicate the region of the prototroch. (**A**) 10–11 days posthatching (dph) larva. (**B**) 3D reconstruction of a confocal scan with volume rendering (blend mode) of autofluorescence of larva (light blue) and surface rendering of reflection signal of expression domain (yellow); 6–11 dph larva in left ventrolateral view with ventral part of autofluorescence signal omitted. Note that the gene expression signal does not extend to the outermost cell layer of the larva. (**C**) Same specimen and reconstruction as in (**B**) in apical/anterior view with apical/anterior part of autofluorescence signal omitted. Note that the gene expression signal is restricted to the subepidermal parts of the trunk and that it is absent in both the calymma and the gut. (D) 3D reconstruction of a confocal scan with volume rendering (blend mode) of autofluorescence of larva (light blue) and surface rendering of reflection signal of expression domain (yellow); 10–11 dph larva in left lateral view with left part of autofluorescence signal omitted. Note the bifurcation of the anterior part of the expression domain with one branch encompassing the foregut (double arrowhead) and another branch extending in anteriodorsal direction (arrow). The gene expression signal does not extend to the outermost cell layer of the larva. (**E**) 14–15 dph larva. Note the ventral fusion of the expression domain (double arrowhead) and the remnants of the lateral bands in the trunk (arrows). (**F**) 12–13 dph larva. (**G**) 3D reconstruction of a confocal scan with volume rendering (blend mode) of autofluorescence of larva (light blue) and surface rendering of reflection signal of expression domain (yellow); 14–15 dph larva in left dorsolateral view with dorsal part of autofluorescence signal omitted. Note that the expression domain encircles the foregut. Remnants of its lateral bands in the trunk are still visible (arrows) and expression is purely subepithelial. (**H**) 3D reconstruction of a confocal scan with volume rendering (blend mode) of autofluorescence of larva (light blue) and surface rendering of reflection signal of expression domain (yellow); 10–11 dph larva in left ventrolateral view with ventral part of autofluorescence signal omitted. Note that remnants of the lateral bands of the expression domain are still visible in the trunk (arrows) and that *twist* expression is purely subepithelial. (**I**) 14–15 dph larva showing *twist* expression domain (arrows) encircling the foregut. (**J**) 3D reconstruction of a confocal scan with volume rendering (MIP (max) mode) of autofluorescence of larva (dark blue) and surface rendering of reflection signal of expression domain (yellow); 14–15 dph larva in ventral view. (**K**) 3D reconstruction of a confocal scan with volume rendering (blend mode) of autofluorescence of larva (light blue) and surface rendering of reflection signal of expression domain (yellow); same specimen as in (**J**) in apical/anterior view with apical/anterior part of autofluorescence signal omitted. Note that *twist* expression is purely subepithelial. (**L**) 16–18 dph juvenile showing *twist* expression domain (arrows) encircling the foregut. pis, peri‐imaginal space; cc, calymma cell; f, foregut; g, gut; t, outgrowing trunk.

**Figure 5:**
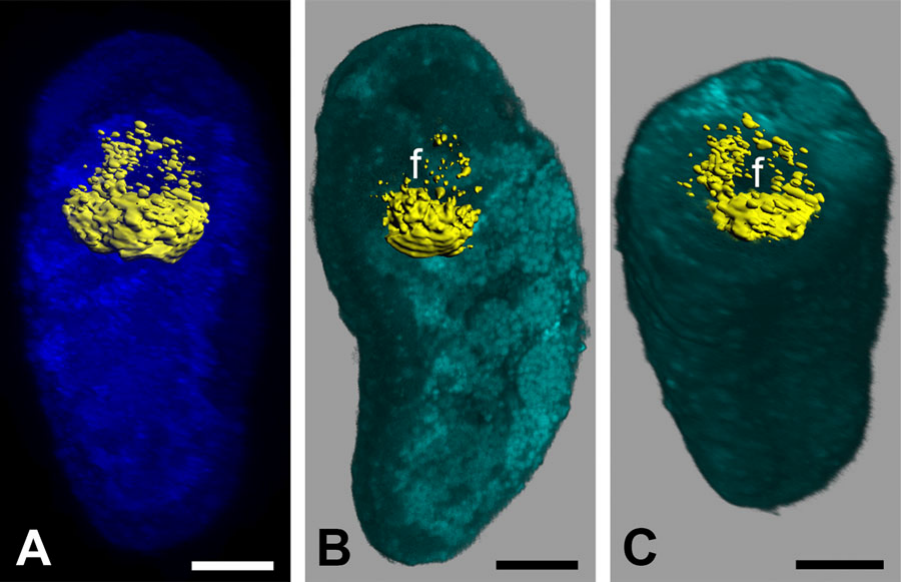
Expression of *twist* in juveniles of *Wirenia argentea*. Anterior is up in (**A**) and (**B**); dorsal is up in (**C**); scale bar equals 20 μm in all panels. (**A**) 3D reconstruction of a confocal scan with volume rendering (MIP (max) mode) of autofluorescence of animal (dark blue) and surface rendering of reflection signal of expression domain (yellow); 17–19 days posthatching (dph) juvenile of *W. argentea* in ventral view. (**B**) 3D reconstruction of a confocal scan with volume rendering (blend mode) of autofluorescence of larva (light blue) and surface rendering of reflection signal of expression domain (yellow); same specimen as in (**A**) in left ventrolateral view with left part of autofluorescence signal omitted. (**C**) Same specimen and reconstruction as in (**B**) in anterioventral view with anterior part of autofluorescence signal omitted. f, foregut.

In newly hatched larvae, *twist* is expressed in a pair of roughly spherical regions to the left and the right of the sagittal plane of the animal (Fig. 3A). These regions mostly show a more or less broad interconnection between them, thus giving the expression domain a slightly peanut‐shaped appearance (Figs. 2A and A′ and 3B–D). 3D reconstructions demonstrate that the signal has a more or less central location and does not extend to the outermost, that is, epidermal cell layer (Fig. 3C and D). Later, the *twist* expression domain becomes more distinctly paired and shifts to the ventral side of the larva but still no signal can be detected in the epidermal cell layer (Fig. 3E–G)—a trait that can be observed throughout development. The expression domain subsequently extends anteriorly, where it encompasses the developing foregut dorsoanteriorly, as well as posteriorly, where it reaches into the developing trunk of the animal (Figs. 2B and B′ and 3H–J). The latter parts of the expression domain elongate as the trunk grows longer and form a pair of lateral bands situated left and right to the sagittal plane with only occasionally a few median interconnections (Fig. 3K and L). The anterior part of the expression domain loses its ventrally fused part (Fig. 3K). This process continues until the *twist* expression domain constitutes a pair of uniform, longitudinal bands (with occasional median interconnections especially at their anterior and near their posterior pole) that run along the major length of the animal (Figs. 2C and 4A and B). In the region between the two bands the gut is situated (Figs. 2C′ and 4C). Hereby, *twist* expression is always exclusively subepithelial, that is, the transcript is neither present in the epidermis—be it in the anterior or the trunk region of the body—nor in the gut (Figs. 3L and 4B and C). Sometimes, the anterior part of the expression domain is bifurcated with one branch encompassing the foregut and another branch extending in an anteriodorsal direction (Fig. 4D). In larvae approaching metamorphosis, the pair of bands in the trunk gradually ceases to express *twist* and its expression is restricted to a paired but often ventrally fused region located ventrally and posteriorly to the foregut, often encircling it anteriorly (Figs. 2D and D′ and 4E–K). Sometimes, remnants of the lateral bands can still be detected (Fig. 4E, G, and H). Again, *twist* expression is exclusively subepithelial (Fig. 4G, H, and K). Shortly before the completion of metamorphosis, the remnants of the lateral bands disappear (Fig. 4I and J). In juvenile animals, *twist* continues to be expressed around the foregut (Figs. 2E and E′, 4L, and 5A–C).

## DISCUSSION

### Mesoderm Formation and Differentiation

Since we found expression of the *twist* ortholog in developmental stages of *W. argentea* exclusively between the body wall and the gut, *twist* is probably expressed as in other bilaterians, that is, mainly in mesodermal cells, and a function in mesoderm differentiation is thus highly likely (see, e.g., Technau and Scholz, 2003). For a considerable period of time, *twist* is thereby expressed in a pair of voluminous, longitudinal regions to the left and right of the anterior–posterior axis, which enlarge as the body of the animal elongates, resembling the classical textbook model of spiralian mesodermal bands. This interpretation is substantiated by our data on cell proliferation during development of *W*. *argentea* and *G. pellucida*, which also show a pair of growing subepithelial bands in partially overlapping position with the *twist* expression domain. Yet, in mollusks, the mesodermal bands have generally been described as mostly less extensive and rather short lived, especially when compared to the situation in annelids (Korschelt and Heider, 1936; Raven, 1966; Fioroni, 1971, 1992; Wanninger and Wollesen, 2015). In the worm‐shaped solenogasters, however, the mesodermal bands seem to acquire larger dimensions and persist for a considerable period of time–a condition, which is more similar to other spiralians, especially annelids (e.g., Mead, 1897; Schmidt, 1925; Okada, 1940; Anderson, 1959, 1966, 1973; Goto et al., ’99; Ackermann et al., 2005; Seaver et al., 2005; Dill et al., 2007; Woodruff et al., 2007; Fischer and Arendt, 2013). Furthermore, the situation we observed is in agreement with classical descriptions on mesoderm formation and differentiation of solenogasters and polyplacophorans (Pruvot, 1890, 1892; Heath, 1899; Naef, 1924; Hammarsten and Runnström, 1925; Baba, 1938; Thompson, 1960; Salvini‐Plawen and Bartolomaeus, 1995) but is demonstrated here for the first time over the entire course of development using state‐of‐the‐art techniques. This suggests that the aculiferan mollusks have retained the plesiomorphic state of extensive and long‐lived mesodermal bands, and that the ephemeral and often rudimentary mesodermal bands are a derived condition of conchiferans (e.g., Patten, 1886; Lillie, 1895; Conklin, 1897; Wierzejski, 1905; Smith, 1935; Okada, 1936 1939; ’Crofts, 1937; Hinman and Degnan, 2002; Le Gouar et al., 2003; Lyons et al., 2012).

*Twist* has an important function in myogenesis in several bilaterian taxa (see, e.g., Castanon and Baylies, 2002 and Technau and Scholz, 2003 for reviews). In comparing our gene expression patterns with data on myogenesis in solenogasters, we did not find *twist* to be expressed in certain developing muscles but rather uniformly in the undifferentiated mesoderm. This is also supported by the fact that myogenesis occurs mainly in stages after *twist* expression has been downregulated to the small elongated areas posterior to the mouth opening (cf. Scherholz et al., 2013, 2015). The reason for this may be that high levels of *twist* expression are required for the specification of muscle progenitor cells and for keeping them in their undifferentiated state, but not for their further differentiation into musculature, as it is the case in the somites of vertebrates and the adult musculature of *Drosophila* (Castanon and Baylies, 2002; Barnes and Firulli, 2009). In the polychaete *Capitella*, *twist* is also downregulated in later stages but in contrast to Solenogastres, it is expressed in the mesodermal bands simultaneously to the development of the musculature for a considerable time (Dill et al., 2007).

Recently, a hypothesis emerged that suggested homology of a ventromedian longitudinal muscle occurring in aculiferan mollusks, annelids, and several other protostomes with the notochord of chordates (Lauri et al., 2014; Brunet et al., 2015). This reasoning was based, among others, on the shared expression of a specific set of genes, including *twist*, in the cells forming this muscular “axochord” in annelids as well as in the developing notochord of chordates. Since we did not find *twist* expression during any stage of development of the ventromedian longitudinal muscle in *W. argentea*, we consider this “axochord hypothesis”—and the proposed homology of the ventromedian longitudinal muscle of protostomes and the chordate notochord—highly unlikely.

### Mollusks and Segmentation

In annelids, the mesodermal bands are separated into distinct subunits—the anlagen of the future segments. Consequently, the pattern of proliferating cells displays a succession of stripes or clusters along the anterior–posterior axis and/or a concentration in the terminal part of the body, thus reflecting segmentation and the existence of a posterior growth zone (de Rosa et al., 2005; Seaver et al., 2005; Brinkmann and Wanninger, 2010). Even some representatives of annelid groups that completely lack segmentation as adults, namely echiurans and sipunculans, show such transient patterns during larval life. In addition, sometimes a general increase in staining intensity of proliferating cells from anterior to posterior can be observed, which mirrors the subsequent differentiation of the body in this direction as is present in those annelids that do exhibit true segments (Hessling, 2003; Kristof et al., 2011). In our study, we were unable to identify such a pattern in the two solenogaster species investigated. Instead, we found an unchanged mode of cell proliferation in the anterior part of the animal and a continuously elongating, paired band of proliferating cells with always homogenous signal intensity over its entire length without any sign of seriality or segmentation, that is, a situation similar to early stages in annelid ontogenesis before the onset of segmentation (cf. Seaver et al., 2005).

The process of segmentation via posterior growth in annelids is also reflected by the expression patterns of the respective *twist* orthologs, which show a pair of uniform bands in earlier stages and a series of stripes or patches along the anterior–posterior axis (usually with a gradient of rising intensity in this direction) in later stages of development (Dill et al., 2007; Pfeifer et al., 2013; Kozin et al., 2016). Again, this is not the case in the solenogaster species we investigated, which, by contrast, consistently shows a rather uniform expression domain in all stages. Our results thus support the notion that the mollusks split off from a common ancestral annelid‐mollusk lineage before segmentation was established. This reasoning is in line with data on *twist* expression in gastropods (Nederbragt et al., 2002; Perry et al., 2015) and other nonsegmented animals such as nemertines, brachiopods, and priapulids (Martín‐Durán and Hejnol, 2015; Martín‐Durán et al., 2015; Passamaneck et al., 2015), as well as with data on the myo‐ and neurogenesis in polyplacophorans and solenogasters (Friedrich et al., 2002; Voronezhskaya et al., 2002; Wanninger and Haszprunar, 2002; Scherholz et al., 2013; Redl et al., 2014), which likewise do not show any sign of rudimentary segmentation during ontogeny.

## CONCLUSIONS

Our data on cell proliferation and *twist* expression during solenogaster development demonstrate the presence of extensive and long‐lived mesodermal bands—a situation that is typical for annelids but within mollusks seems to be restricted to the aculiferans, while the conchiferans show rather rudimentary and ephemeral mesodermal bands. In contrast to annelids, however, both the pattern of proliferating cells and the *twist* expression domain—and thus the mesodermal bands—do not show a separation into distinct serial subunits. This configuration clearly argues against a segmented ancestry of mollusks. The lack of *twist* expression during ventromedian muscle formation in solenogasters strongly argues against homology of such a protostomian muscle with the notochord of chordates.

## AUTHORS’ CONTRIBUTIONS

E.R. performed the experiments, analyzed the data, and drafted the manuscript. M.S. designed the primers, amplified and cloned the *twist* fragment, and synthesized the probe for *in situ* hybridization. E.R., M.S., and T.W. extracted the RNA and synthesized the cDNA. E.R. and T.W. performed the bioinformatics analysis. E.R., M.S., C.T., and T.W. reared the study material. C.T. coordinated and supervised research in Bergen. A.W. designed the study, supervised the project, and contributed to data interpretation and writing of the manuscript. All authors provided input and read and approved the final version of the manuscript.

## Supporting information


**Supplementary Figure S1**. Orthology assessment of *twist* of *Wirenia argentea*. (A) Alignment of partial twist amino acid sequences showing the common bHLH domain and the WR motif characteristic for twist. (B) Neighbor‐Joining Tree showing the three well supported clusters for paraxis, dHAND, and twist. Bootstrap values higher than 50% (10,000 replicates) are indicated at the nodes. Abbreviations: Am, *Apis mellifera*; Av, *Alitta virens*; Cf, *Crepidula fornicata*; Dr, *Danio rerio*; Lv, *Lytechinus variegatus*; Mm, *Mus musculus*; Na, *Novocrania anomala*; Pc, *Priapulus caudatus*; Pd, *Platynereis dumerilii*; Ph, *Parhyale hawaiensis*; Pod, *Podocoryna carnea*; Pv, *Patella vulgata*; Sk, *Saccoglossus kowalevskii*; Sp, *Strongylocentrotus purpuratus*; Tc, *Tribolium castaneum*; Tt, *Terebratalia transversa*; War, *Wirenia argentea*; Xl, *Xenopus laevis*.

## References

[jezb22714-bib-0001] Abouheif E . 1997 Developmental genetics and homology: a hierarchical approach. Trends Ecol Evol 12:405–408. 21238133 10.1016/s0169-5347(97)01125-7

[jezb22714-bib-0002] Abouheif E , Akam M , Dickinson WJ , et al. 1997 Homology and developmental genes. Trends Genet 13:432–433. 9385839 10.1016/s0168-9525(97)01271-7

[jezb22714-bib-0003] Ackermann C , Dorresteijn A , Fischer A . 2005 Clonal domains in postlarval *Platynereis dumerilii* (Annelida: Polychaeta). J Morphol 266:258–280. 16170805 10.1002/jmor.10375

[jezb22714-bib-0004] Anderson DT . 1959 The embryology of the polychaete *Scoloplos armiger* . Q J Micr Sci 100:89–166.

[jezb22714-bib-0005] Anderson DT . 1966 The comparative embryology of the Polychaeta. Acta Zool 47:1–42.

[jezb22714-bib-0006] Anderson DT . 1973 Embryology and phylogeny in annelids and arthropods. Oxford: Pergamon Press.

[jezb22714-bib-0007] Baba K . 1938 The later development of a solenogastre, *Epimenia verrucosa* (nierstrasz). J Dept Agric Kyūsyū Imp Univ 6:21–40.

[jezb22714-bib-0008] Balavoine G . 2014 Segment formation in annelids: patterns, processes and evolution. Int J Dev Biol 58:469–483. 25690963 10.1387/ijdb.140148gb

[jezb22714-bib-0009] Balavoine G , Adoutte A . 2003 The segmented *Urbilateria*: a testable scenario. Integr Comp Biol 43:137–147. 21680418 10.1093/icb/43.1.137

[jezb22714-bib-0010] Barnes RM , Firulli AB . 2009 A twist of insight—the role of Twist‐family bHLH factors in development. Int J Dev Biol 53:909–924. 19378251 10.1387/ijdb.082747rb PMC2737731

[jezb22714-bib-0011] Blair SS . 2008 Segmentation in animals. Curr Biol 18:R991–R995. 19000806 10.1016/j.cub.2008.08.029

[jezb22714-bib-0012] Bolger AM , Lohse M , Usadel B . 2014 Trimmomatic: a flexible trimmer for Illumina sequence data. Bioinformatics 30:2114–2120. 24695404 10.1093/bioinformatics/btu170 PMC4103590

[jezb22714-bib-0013] Brinkmann N , Wanninger A . 2010 Integrative analysis of polychaete ontogeny: cell proliferation patterns and myogenesis in trochophore larvae of *Sabellaria alveolata* . Evol Dev 12:5–15. 20156278 10.1111/j.1525-142X.2009.00386.x

[jezb22714-bib-0014] Brunet T , Lauri A , Arendt D . 2015 Did the notochord evolve from an ancient axial muscle? The axochord hypothesis. BioEssays 37:836–850. 26172338 10.1002/bies.201500027 PMC5054868

[jezb22714-bib-0015] Castanon I , Baylies MK . 2002 A twist in fate: evolutionary comparison of twist structure and function. Gene 287:11–22. 11992718 10.1016/s0378-1119(01)00893-9

[jezb22714-bib-0016] Cavanagh BL , Walker T , Norazit A , Meedeniya ACB . 2011 Thymidine analogues for tracking DNA synthesis. Molecules 16:7980–7993. 21921870 10.3390/molecules16097980 PMC6264245

[jezb22714-bib-0017] Chipman AD . 2010 Parallel evolution of segmentation by co‐option of ancestral gene regulatory networks. BioEssays 32:60–70. 20020480 10.1002/bies.200900130

[jezb22714-bib-0018] Conklin EG . 1897 The embryology of *Crepidula*, a contribution to the cell lineage and early development of some marine gasteropods. J Morphol 13:1–226.

[jezb22714-bib-0019] Couso JP . 2009 Segmentation, metamerism and the Cambrian explosion. Int J Dev Biol 53:1305–1316. 19247939 10.1387/ijdb.072425jc

[jezb22714-bib-0020] Crofts DR . 1937 The development of *Haliotis tuberculata*, with special reference to organogenesis during torsion. Philos Trans R Soc Lond B Biol Sci 228:219–268.

[jezb22714-bib-0021] De Rosa R , Prud'homme B , Balavoine G . 2005 *caudal* and *even‐skipped* in the annelid *Platynereis dumerilii* and the ancestry of posterior growth. Evol Dev 7:574–587. 16336411 10.1111/j.1525-142X.2005.05061.x

[jezb22714-bib-0022] Dill KK , Thamm K , Seaver EC . 2007 Characterization of *twist* and *snail* gene expression during mesoderm and nervous system development in the polychaete annelid *Capitella* sp. I. Dev Genes Evol 217:435–447. 17473935 10.1007/s00427-007-0153-4

[jezb22714-bib-0023] Erwin DH , Davidson EH . 2002 The last common bilaterian ancestor. Development 129:3021–3032. 12070079 10.1242/dev.129.13.3021

[jezb22714-bib-0024] Fioroni P . 1971 Die Entwicklungstypen der Mollusken, eine vergleichend‐embryologische Studie. Z wiss Zool 182:263–394.

[jezb22714-bib-0025] Fioroni P . 1992 Allgemeine und vergleichende Embryologie der Tiere. Berlin, Heidelberg: Springer Verlag.

[jezb22714-bib-0026] Fischer AHL , Arendt D . 2013 Mesoteloblast‐like mesodermal stem cells in the polychaete annelid *Platynereis dumerilii* (Nereididae). J Exp Zool B (Mol Dev Evol) 320:94–104. 23408594 10.1002/jez.b.22486

[jezb22714-bib-0027] Fischer AHL , Henrich T , Arendt D . 2010 The normal development of *Platynereis dumerilii* (Nereididae, Annelida). Front Zool 7:31. 21192805 10.1186/1742-9994-7-31 PMC3027123

[jezb22714-bib-0028] Friedrich S , Wanninger A , Brückner M , Haszprunar G . 2002 Neurogenesis in the mossy chiton, *Mopalia muscosa* (Gould) (Polyplacophora): evidence against molluscan metamerism. J Morphol 253:109–117. 12112126 10.1002/jmor.10010

[jezb22714-bib-0029] Fritsch M , Wollesen T , de Oliveira AL , Wanninger A . 2015 Unexpected co‐linearity of Hox gene expression in an aculiferan mollusk. BMC Evol Biol 15:151. 26243538 10.1186/s12862-015-0414-1 PMC4524011

[jezb22714-bib-0030] Goto A , Kitamura K , Shimizu T . 1999 Cell lineage analysis of pattern formation in the *Tubifex* embryo. I. Segmentation in the mesoderm. Int J Dev Biol 43:317–327. 10470648

[jezb22714-bib-0031] Gurevich A , Saveliev V , Vyahhi N , Tesler G . 2013 QUAST: quality assessment tool for genome assemblies. Bioinformatics 29:1072–1075. 23422339 10.1093/bioinformatics/btt086 PMC3624806

[jezb22714-bib-0032] Hammarsten OD , Runnström J . 1925 Zur Embryologie von *Acanthochiton discrepans* brown . Zool Jahrb Abt Anat 47:261–318.

[jezb22714-bib-0033] Handel K , Basal A , Fan X , Roth S . 2005 *Tribolium castaneum twist*: gastrulation and mesoderm formation in a short‐germ beetle. Dev Genes Evol 215:13–31. 15645317 10.1007/s00427-004-0446-9

[jezb22714-bib-0034] Heath H . 1899 The development of *Ischnochiton* . Zool Jahrb Abt Anat 12:567–656.

[jezb22714-bib-0035] Hessling R . 2002 Metameric organisation of the nervous system in developmental stages of *Urechis caupo* (Echiura) and its phylogenetic implications. Zoomorphology 121:221–234.

[jezb22714-bib-0036] Hessling R . 2003 Novel aspects of the nervous system of *Bonellia viridis* (Echiura) revealed by the combination of immunohistochemistry, confocal laser‐scanning microscopy and three‐dimensional reconstruction. Hydrobiologia 496:225–239.

[jezb22714-bib-0037] Hessling R , Westheide W . 2002 Are Echiura derived from a segmented ancestor? Immunohistochemical analysis of the nervous system in developmental stages of *Bonellia viridis* . J Morphol 252:100–113. 11921039 10.1002/jmor.1093

[jezb22714-bib-0038] Hinman VF , Degnan BM . 2002 *Mox* homeobox expression in muscle lineage of the gastropod *Haliotis asinina*: evidence for a conserved role in bilaterian myogenesis. Dev Genes Evol 212:141–144. 11976952 10.1007/s00427-002-0223-6

[jezb22714-bib-0039] Jékely G , Arendt D . 2007 Cellular resolution expression profiling using confocal detection of NBT/BCIP precipitate by reflection microscopy. BioTechniques 42:751–755. 17612299 10.2144/000112462

[jezb22714-bib-0040] Kocot KM , Cannon JT , Todt C , et al. 2011 Phylogenomics reveals deep molluscan relationships. Nature 477:452–456. 21892190 10.1038/nature10382 PMC4024475

[jezb22714-bib-0041] Korschelt E , Heider K . 1936 Vergleichende Entwicklungsgeschichte der Tiere. Jena: Verlag von Gustav Fischer.

[jezb22714-bib-0042] Kozin VV , Filimonova DA , Kupriashova EE , Kostyuchenko RP . 2016 Mesoderm patterning and morphogenesis in the polychaete *Alitta virens* (Spiralia, Annelida): expression of mesodermal markers *twist*, *mox*, *evx* and functional role for MAP kinase signaling. Mech Dev 140:1–11. 27000638 10.1016/j.mod.2016.03.003

[jezb22714-bib-0043] Kristof A , Wollesen T , Wanninger A . 2008 Segmental mode of neural patterning in Sipuncula. Curr Biol 18:1129–1132. 18656359 10.1016/j.cub.2008.06.066

[jezb22714-bib-0044] Kristof A , Wollesen T , Maiorova AS , Wanninger A . 2011 Cellular and muscular growth patterns during sipunculan development. J Exp Zool B (Mol Dev Evol) 316:227–240. 10.1002/jez.b.21394 PMC4682194 21246707

[jezb22714-bib-0045] Lauri A , Brunet T , Handberg‐Thorsager M , et al. 2014 Development of the annelid axochord: insights into notochord evolution. Science 345:1365–1368. 25214631 10.1126/science.1253396

[jezb22714-bib-0046] Le Gouar M , Lartillot N , Adoutte A , Vervoort M . 2003 The expression of a *caudal* homologue in a mollusc, *Patella vulgata* . Gene Expr Patterns 3:35–37. 12609599 10.1016/s1567-133x(02)00091-1

[jezb22714-bib-0047] Lillie FR . 1895 The embryology of the Unionidae. A study in cell‐lineage. J Morphol 10:1–100.

[jezb22714-bib-0048] Lyons DC , Perry KJ , Lesoway MP , Henry JQ . 2012 Cleavage pattern and fate map of the mesentoblast, 4d, in the gastropod *Crepidula*: a hallmark of spiralian development. EvoDevo 3:21. 22992254 10.1186/2041-9139-3-21 PMC3724503

[jezb22714-bib-0049] Martín‐Durán JM , Hejnol A . 2015 The study of *Priapulus caudatus* reveals conserved molecular patterning underlying different gut morphogenesis in the Ecdysozoa. BMC Biol 13:29. 25895830 10.1186/s12915-015-0139-z PMC4434581

[jezb22714-bib-0050] Martín‐Durán JM , Vellutini BC , Hejnol A . 2015 Evolution and development of the adelphophagic, intracapsular Schmidt's larva of the nemertean *Lineus ruber* . EvoDevo 6:28. 26417429 10.1186/s13227-015-0023-5 PMC4584431

[jezb22714-bib-0051] Mead AD . 1897 The early development of marine annelids. J Morphol 13:227–326.

[jezb22714-bib-0052] Minelli A . 2015 EvoDevo and its significance for animal evolution and phylogeny In: Wanninger A, editor. Evolutionary developmental biology of invertebrates, Volume 1: introduction, non‐bilateria, Acoelomorpha, Xenoturbellida, Chaetognatha. Vienna: Springer‐Verlag p 1–23.

[jezb22714-bib-0053] Minelli A , Fusco G . 2013 Homology In: Kampourakis K, editor. The philosophy of biology. A companion for educators. Dordrecht: Springer p 289–322.

[jezb22714-bib-0054] Naef A. 1924 Studien zur generellen Morphologie der Mollusken. 3. Teil: Die typischen Beziehungen der Weichtierklassen untereinander und das Verhältnis ihrer Urformen zu anderen Cölomaten. Erg Fortschr Zool 6:27–124.

[jezb22714-bib-0055] Nederbragt AJ , Lespinet O, van Wageningen S, et al . 2002 A lophotrochozoan *twist* gene is expressed in the ectomesoderm of the gastropod mollusk *Patella vulgata* . Evol Dev 4:334–343. 12356263 10.1046/j.1525-142x.2002.02020.x

[jezb22714-bib-0056] Odhner NH . 1921 Norwegian Solenogastres. Bergens Mus Aarb 1918‐19 Naturvid række 3:1–86.

[jezb22714-bib-0057] Okada K . 1936 Some notes on *Sphaerium japonicum biwaense* mori, a freshwater bivalve. IV. Gastrula and fetal larva. Sci Rep Tôhoku Imp Univ Ser IV 11:49–68.

[jezb22714-bib-0058] Okada K . 1939 The development of the primary mesoderm in *Sphaerium japonicum biwaense* mori. Sci Rep Tôhoku Imp Univ Ser IV 14:25–48.

[jezb22714-bib-0059] Okada K . 1940 The gametogenesis, the breeding habits, and the early development of *Arenicola cristata* stimpson, a tubicolous polychaete. Sci Rep Tôhoku Imp Univ Ser IV 16:99–146.

[jezb22714-bib-0060] Passamaneck YJ , Hejnol A , Martindale MQ . 2015 Mesodermal gene expression during the embryonic and larval development of the articulate brachiopod *Terebratalia transversa* . EvoDevo 6:10. 25897375 10.1186/s13227-015-0004-8 PMC4404124

[jezb22714-bib-0061] Patten W . 1886 The embryology of *Patella* . Arb Zool Inst Univ Wien u Zool Stat Triest 6:149–174.

[jezb22714-bib-0062] Peng Y , Leung HCM , Yiu S‐M , et al. 2013 IDBA‐tran: a more robust de novo de Bruijn graph assembler for transcriptomes with uneven expression levels. Bioinformatics 29:i326–i334. 23813001 10.1093/bioinformatics/btt219 PMC3694675

[jezb22714-bib-0063] Perry KJ , Lyons DC , Truchado‐Garcia M , et al. 2015 Deployment of regulatory genes during gastrulation and germ layer specification in a model spiralian mollusc *Crepidula* . Dev Dyn 244:1215–1248. 26197970 10.1002/dvdy.24308

[jezb22714-bib-0064] Pfeifer K , Schaub C , Wolfstetter G , Dorresteijn A . 2013 Identification and characterization of a *twist* ortholog in the polychaete annelid *Platynereis dumerilii* reveals mesodermal expression of *Pdu‐twist* . Dev Genes Evol 223:319–328. 23817621 10.1007/s00427-013-0448-6

[jezb22714-bib-0065] Price AL , Patel NH . 2008 Investigating divergent mechanisms of mesoderm development in arthropods: The expression of *Ph‐twist* and *Ph‐mef 2* in *Parhyale hawaiensis* . J Exp Zool B (Mol Dev Evol) 310:24–40. 17152085 10.1002/jez.b.21135

[jezb22714-bib-0066] Pruvot G . 1890 Sur le développement d'un solénogastre. C R hebd Séances Acad Sci 111:689–692.

[jezb22714-bib-0067] Pruvot G . 1892 Sur l'embryogénie d'une *Proneomenia* . C R hebd Séances Acad Sci 114:1211–1214.

[jezb22714-bib-0068] Ratnasingham S , Hebert PDN . 2007 BOLD: the Barcode of Life Data System (www.barcodinglife.org). Mol Ecol Notes 7:355–364. 18784790 10.1111/j.1471-8286.2007.01678.x PMC1890991

[jezb22714-bib-0069] Raven CP . 1966 Morphogenesis. The analysis of molluscan development. Oxford: Pergamon Press.

[jezb22714-bib-0070] Redl E , Scherholz M , Todt C , Wollesen T , Wanninger A . 2014 Development of the nervous system in Solenogastres (Mollusca) reveals putative ancestral spiralian features. EvoDevo 5:48. 25904999 10.1186/2041-9139-5-48 PMC4406162

[jezb22714-bib-0071] Salvini‐Plawen Lv . 1972 Zur Morphologie und Phylogenie der Mollusken: Die Beziehungen der Caudofoveata und der Solenogastres als Aculifera, als Mollusca und als Spiralia (nebst einem Beitrag zur Phylogenie der coelomatischen Räume). Z wiss Zool 184:205–394.

[jezb22714-bib-0072] Salvini‐Plawen Lv . 1980 Was ist eine Trochophora? Eine Analyse der Larventypen mariner Protostomier. Zool Jahrb Abt Anat 103:389–423.

[jezb22714-bib-0073] Salvini‐Plawen Lv . 2003 On the phylogenetic significance of the aplacophoran Mollusca. Iberus 21:67–97.

[jezb22714-bib-0074] Salvini‐Plawen Lv , Bartolomaeus T . 1995 Mollusca: mesenchymata with a “coelom” In: Lanzavecchia G, Valvassori R, Candia Carnevali MD, editors. Body cavities: function and phylogeny. Modena: Mucchi p 75–92.

[jezb22714-bib-0075] Scherholz M , Redl E , Wollesen T , Todt C , Wanninger A . 2013 Aplacophoran mollusks evolved from ancestors with polyplacophoran‐like features. Curr Biol 23:2130–2134. 24139743 10.1016/j.cub.2013.08.056 PMC3898471

[jezb22714-bib-0076] Scherholz M , Redl E , Wollesen T , Todt C , Wanninger A . 2015 From complex to simple: myogenesis in an aplacophoran mollusk reveals key traits in aculiferan evolution. BMC Evol Biol 15:201. 26385077 10.1186/s12862-015-0467-1 PMC4575435

[jezb22714-bib-0077] Schmidt GA . 1925 Untersuchungen über die Embryologie der Anneliden. I. Die Embryonalentwicklung von *Piscicola geometra* blainv . Zool Jahrb Abt Anat 47:319–428.

[jezb22714-bib-0078] Seaver EC . 2003 Segmentation: mono‐ or polyphyletic? Int J Dev Biol 47:583–595. 14756334

[jezb22714-bib-0079] Seaver EC , Thamm K , Hill SD . 2005 Growth patterns during segmentation in the two polychaete annelids, *Capitella* sp. I and *Hydroides elegans*: comparisons at distinct life history stages. Evol Dev 7:312–326. 15982368 10.1111/j.1525-142X.2005.05037.x

[jezb22714-bib-0080] Shimizu T , Nakamoto A . 2001 Segmentation in annelids: cellular and molecular basis for metameric body plan. Zool Sci 18:285–298.

[jezb22714-bib-0081] Smith FGW . 1935 The development of *Patella vulgata* . Philos Trans R Soc Lond B Biol Sci 225:95–125.

[jezb22714-bib-0082] Smith SA , Wilson NG , Goetz FE , et al. 2011 Resolving the evolutionary relationships of molluscs with phylogenomic tools. Nature 480:364–367. 22031330 10.1038/nature10526

[jezb22714-bib-0083] Smith SA , Wilson NG , Goetz FE , et al. 2013 Corrigendum: resolving the evolutionary relationships of molluscs with phylogenomic tools. Nature 493:708. 10.1038/nature10526 22031330

[jezb22714-bib-0084] Sommer RJ , Tautz D . 1994 Expression patterns of *twist* and *snail* in *Tribolium* (Coleoptera) suggest a homologous formation of mesoderm in long and short germ band insects. Dev Genet 15:32–37. 8187348 10.1002/dvg.1020150105

[jezb22714-bib-0085] Spring J , Yanze N , Middel AM , Stierwald M , Gröger H , Schmid V . 2000 The mesoderm specification factor twist in the life cycle of jellyfish. Dev Biol 228:363–375. 11112336 10.1006/dbio.2000.9956

[jezb22714-bib-0086] Tautz D . 2004 Segmentation. Dev Cell 7:301–312. 15363406 10.1016/j.devcel.2004.08.008

[jezb22714-bib-0087] Tavares AT , Izpisúa‐Belmonte JC , Rodríguez‐León J . 2001 Developmental expression of chick *twist* and its regulation during limb patterning. Int J Dev Biol 45:707–713. 11669372

[jezb22714-bib-0088] Technau U , Scholz CB . 2003 Origin and evolution of endoderm and mesoderm. Int J Dev Biol 47:531–539. 14756329

[jezb22714-bib-0089] Thompson TE . 1960 The development of *Neomenia carinata* Tullberg (Mollusca Aplacophora). Proc R Soc B 153:263–278.

[jezb22714-bib-0090] Vinther J , Sperling EA , Briggs DEG , Peterson KJ . 2012 A molecular palaeobiological hypothesis for the origin of aplacophoran molluscs and their derivation from chiton‐like ancestors. Proc R Soc B 279:1259–1268. 10.1098/rspb.2011.1773 PMC3282371 21976685

[jezb22714-bib-0091] Voronezhskaya EE , Tyurin SA , Nezlin LP . 2002 Neuronal development in larval chiton *Ischnochiton hakodadensis* (Mollusca: Polyplacophora). J Comp Neurol 444:25–38. 11835180 10.1002/cne.10130

[jezb22714-bib-0092] Wagner GP . 2014 Homology, genes, and evolutionary innovation. Princeton, NJ: Princeton University Press.

[jezb22714-bib-0093] Wagner GP . 2015 Homology in the age of developmental genomics In: Wanninger A, editor. Evolutionary developmental biology of invertebrates, Volume 1: introduction, non‐bilateria, Acoelomorpha, Xenoturbellida, Chaetognatha. Vienna: Springer‐Verlag p 25–43.

[jezb22714-bib-0094] Wanninger A , Haszprunar G . 2002 Chiton myogenesis: perspectives for the development and evolution of larval and adult muscle systems in molluscs. J Morphol 251:103–113. 11748697 10.1002/jmor.1077

[jezb22714-bib-0095] Wanninger A , Wollesen T . 2015 Mollusca In: Wanninger A, editor. Evolutionary developmental biology of invertebrates, Vol. 2: Lophotrochozoa (Spiralia). Vienna: Springer‐Verlag p 103–153.

[jezb22714-bib-0096] Wanninger A , Kristof A , Brinkmann N . 2009 Sipunculans and segmentation. Comm Integr Biol 2:56–59. 10.4161/cib.2.1.7505 PMC2649304 19513266

[jezb22714-bib-0097] Wierzejski A . 1905 Embryologie von *Physa fontinalis* L. Z wiss Zool 83:502–706.

[jezb22714-bib-0098] Woodruff JB , Mitchell BJ , Shankland M . 2007 *Hau‐Pax3/7A* is an early marker of leech mesoderm involved in segmental morphogenesis, nephridial development, and body cavity formation. Dev Biol 306:824–837. 17433288 10.1016/j.ydbio.2007.03.002

[jezb22714-bib-0099] Wray GA , Abouheif E . 1998 When is homology not homology? Curr Opin Genet Dev 8:675–680. 9914205 10.1016/s0959-437x(98)80036-1

[jezb22714-bib-0100] Yamazaki K , Akiyama‐Oda Y , Oda H . 2005 Expression patterns of a *twist*‐related gene in embryos of the spider *Achaearanea tepidariorum* reveal divergent aspects of mesoderm development in the fly and spider. Zool Sci 22:177–185. 15738638 10.2108/zsj.22.177

