# Supplementary figures and images for: Cell Proliferation Pattern and Twist Expression in an Aplacophoran Mollusk Argue Against Segmented Ancestry of Mollusca
Source: J Exp Zool B Mol Dev Evol. 2016 Dec 14;326(7):422–36. doi: 10.1002/jez.b.22714 (PMC5299467; doi:10.1002/jez.b.22714)

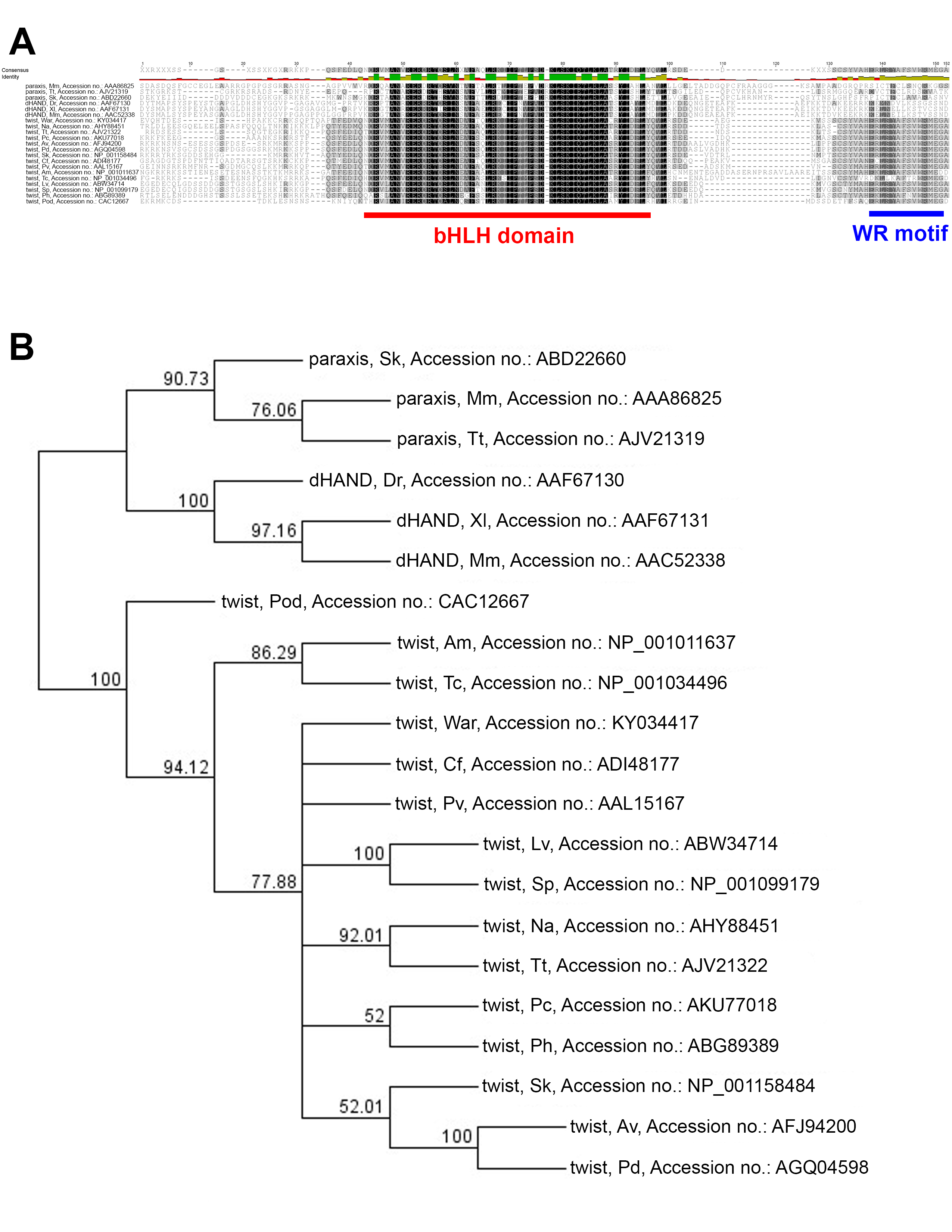

Supplement: Supplementary file 1 — Supplementary Figure S1. Orthology assessment of twist of Wirenia argentea. (A) Alignment of partial twist amino acid sequences showing the common bHLH domain and the WR motif characteristic for twist. (B) Neighbor‐Joining Tree showing the three well supported clusters for paraxis, dHAND, and twist. Bootstrap values higher than 50% (10,000 replicates) are indicated at the nodes. Abbreviations: Am, Apis mellifera; Av, Alitta virens; Cf, Crepidula fornicata; Dr, Danio rerio; Lv, Lytechinus variegatus; Mm, Mus musculus; Na, Novocrania anomala; Pc, Priapulus caudatus; Pd, Platynereis dumerilii; Ph, Parhyale hawaiensis; Pod, Podocoryna carnea; Pv, Patella vulgata; Sk, Saccoglossus kowalevskii; Sp, Strongylocentrotus purpuratus; Tc, Tribolium castaneum; Tt, Terebratalia transversa; War, Wirenia argentea; Xl, Xenopus laevis. [file JEZ-326-422-s001.tif]
